# Differential Gene Expression and Protein Phosphorylation as Factors Regulating the State of the Arabidopsis SNX1 Protein Complexes in Response to Environmental Stimuli

**DOI:** 10.3389/fpls.2016.01456

**Published:** 2016-09-26

**Authors:** Tzvetina Brumbarova, Rumen Ivanov

**Affiliations:** Institute of Botany, Heinrich-Heine UniversityDüsseldorf, Germany

**Keywords:** protein sorting, SNX1, gene expression, phosphorylation, auxin, iron deficiency

## Abstract

Endosomal recycling of plasma membrane proteins contributes significantly to the regulation of cellular transport and signaling processes. Members of the Arabidopsis (*Arabidopsis thaliana*) SORTING NEXIN (SNX) protein family were shown to mediate the endosomal retrieval of transporter proteins in response to external challenges. Our aim is to understand the possible ways through which external stimuli influence the activity of SNX1 in the root. Several proteins are known to contribute to the function of SNX1 through direct protein–protein interaction. We, therefore, compiled a list of all Arabidopsis proteins known to physically interact with SNX1 and employed available gene expression and proteomic data for a comprehensive analysis of the transcriptional and post-transcriptional regulation of this interactome. The genes encoding SNX1-interaction partners showed distinct expression patterns with some, like *FAB1A*, being uniformly expressed, while others, like *MC9* and *BLOS1*, were expressed in specific root zones and cell types. Under stress conditions known to induce SNX1-dependent responses, two genes encoding SNX1-interacting proteins, *MC9* and *NHX6*, showed major gene-expression variations. We could also observe zone-specific transcriptional changes of *SNX1* under iron deficiency, which are consistent with the described role of the SNX1 protein. This suggests that the composition of potential SNX1-containing protein complexes in roots is cell-specific and may be readjusted in response to external stimuli. On the level of post-transcriptional modifications, we observed stress-dependent changes in the phosphorylation status of SNX1, FAB1A, and CLASP. Interestingly, the phosphorylation events affecting SNX1 interactors occur in a pattern which is largely complementary to transcriptional regulation. Our analysis shows that transcriptional and post-transcriptional regulation play distinct roles in SNX1-mediated endosomal recycling under external stress.

## Introduction

The plasma membrane serves as a cellular border and, thanks to membrane-embedded proteins, it is responsible for selectively permitting signals and material inward and outward. The movement of these transmembrane proteins is restricted by their membrane environment and, therefore, complex endomembrane trafficking pathways are responsible for their delivery to the plasma membrane (Reyes et al., 2011; Luschnig and Vert, 2014). The availability of the membrane-embedded proteins at the plasma membrane is a result of rapid cycles of exocytosis and endocytosis to endosomal compartments. Endocytosed proteins are transported to the early endosome/*trans*-Golgi network (EE/TGN; Dhonukshe et al., 2007; Viotti et al., 2010; Barberon et al., 2011; Kasai et al., 2011) and are then being processed in a sorting compartment (Jaillais et al., 2008). There, a decision is made whether proteins should be reset (reverted to their initial inactive state; in the case of active/phosphorylated receptor kinases) and recycled, or sent to the vacuole for degradation (Geldner and Robatzek, 2008). There are several factors known to be required for successful recycling. One of these is the pH change in the endosomal lumen driven by vacuolar H^+^-ATPases (V-ATPases; Dettmer et al., 2006; Kane, 2006; Huang and Chang, 2011). In plants, the EE/TGN-localized V-ATPase is essential for the regulation of secretion and recycling of endosomal proteins such as the brassinosteroid receptor BRI1 and the cellulose synthase A complexes (Luo et al., 2015). In Arabidopsis, the function of the Na^+^/H^+^ antiporters NHX5 and NHX6, which localize to the Golgi apparatus and EE/TGN and export luminal H^+^, was shown to be critical for the vacuolar targeting and recycling of proteins (Bassil et al., 2011; Ashnest et al., 2015). At present, the direct consequence of the pH change remains unexplained. However, it is speculated that it might affect the recruitment of trafficking regulators (Luo et al., 2015). In animals, the recruitment of the small GTPase Arf6 and its GDP/GTP exchange factor ARNO is dependent on the intravesicle pH-change through the interaction with the V-ATPase subunit a2. Thus, V-ATPase components might serve as a pH sensor (Maranda et al., 2001; Hurtado-Lorenzo et al., 2006).

The Arabidopsis ARF-guanine nucleotide exchange factor GNOM is required for the recycling of PIN-family auxin transporters at the EE/TGN (Geldner et al., 2003, 2004). However, GNOM was recently found to mainly localize to subdomains of the Golgi apparatus, indicating that its effect on protein recycling might be indirect (Naramoto et al., 2014).

In animal cells and yeast, the recycling from the sorting compartments requires the action of the retromer complex. The retromer is a multimer complex consisting of two smaller complexes—the core retromer, composed of the proteins VACUOLAR PROTEIN SORTING 26 (VPS26), VPS29, and VPS35, and the SORTING NEXIN (SNX) subcomplex, which consists of homo- or heterodimers of different SNX proteins and, therefore, provides the retromer with versatile functions (Cullen and Korswagen, 2012). In plants, there are homologs of the core retromer subunits. However, in comparison to the more than 30 human SNX proteins, Arabidopsis has only six potential ones, where only three, SNX1, SNX2a, and SNX2b, have been characterized (Zelazny et al., 2012). A major problem in understanding protein sorting in plants is the controversial data on the localization of the retromer components and, therefore, the not entirely clear physical identity of the sorting endosome (Robinson et al., 2012). In some studies, members of the retromer were found to predominantly colocalize with markers for the late endosomes and to a lesser extent with EE/TGN markers (Jaillais et al., 2006, 2007; Oliviusson et al., 2006; Kleine-Vehn et al., 2008; Yamazaki et al., 2008; Ivanov and Gaude, 2009; Pourcher et al., 2010; Nodzynski et al., 2013; Zelazny et al., 2013; Ivanov et al., 2014). Other authors reported a predominant TGN localization, suggesting that the retrograde transport occurs not in the direction multivesicular body (MVB)-to-TGN but rather within TGN itself, or even between TGN and the Golgi apparatus (Niemes et al., 2010; Stierhof et al., 2012). Indirect evidence also supports a TGN localization of the sorting compartment since the Arabidopsis Rab-A2 and Rab-A3 proteins, homologs of the mammalian recycling endosome marker rab11, label the TGN in Arabidopsis roots (Chow et al., 2008). The matter is still heavily debated. However, the analysis of all available data leaves the impression that more than one of the visually detectable plant endosomal compartments is capable of performing recycling functions. Therefore, the term “sorting endosome” probably reflects several stages of endosome maturation (Robinson and Neuhaus, 2016).

At present, the exact composition of the functional retromer complex in Arabidopsis is still unclear. A genetic interaction between SNX1 and the core retromer subunit VPS29 has been demonstrated (Jaillais et al., 2007). At the same time, however, loss-of-function mutants of the core retromer components display severe developmental defects, while knocking out all of the three *SNX* genes results in only mild developmental phenotypes. This suggests that the two subcomplexes function to a large extent independently from each other (Jaillais et al., 2006; Shimada et al., 2006; Yamazaki et al., 2008; Pourcher et al., 2010; Zelazny et al., 2013). The biological significance of SNX for endomembrane trafficking of plasma membrane proteins is revealed by challenging *snx* mutant plants with external stimuli. In wild type plants, gravitropic stimulation, auxin treatment, or high temperature lead to the active retrieval of PIN2 from endosomal compartments (Jaillais et al., 2006; Kleine-Vehn et al., 2008; Hanzawa et al., 2013). In a similar manner, depletion of iron from the root environment causes the synthesis and active recycling of the IRON-REGULATED TRANSPORTER1 (IRT1; Ivanov et al., 2014). In the absence of functional SNX proteins, the recycling of these two transporters is compromised and they are mistargeted to the vacuole, instead. This suggests that the SNX proteins may represent a potential stress-response trafficking module in plant cells, required upon a challenge-driven enhanced demand of specific protein recycling.

Several proteins are known to interact with SNX1 and affect its sorting function and, therefore, changes in the composition of the SNX1 protein complexes might affect protein recycling. Based on this, our aim was to outline the mechanisms through which stress conditions might influence the SNX1 activity, either directly or by modulating the availability or the state of SNX1 itself and its potential interactors. We compiled a complete list of SNX1-interacting proteins and employed the available gene expression and proteomic data for a comprehensive analysis of the transcriptional and post-transcriptional regulation of SNX1, together with all Arabidopsis proteins known to physically interact with it. The data showed distinct expression patterns of genes and specific responses to stress, suggesting that the SNX1-complexes composition in roots is cell-specific and may be readjusted in response to external stimuli. We found that the differential phosphorylation of SNX1-complex members under stress occurs in a pattern complementary to transcriptional regulation. Our analysis shows that the efficiency of SNX1-mediated protein sorting under stress conditions may be influenced by a complementary action of transcriptional and post-transcriptional signals targeting different components of the SNX1 interactome.

## Materials and Methods

### SNX1 Interactome Prediction

The Arabidopsis SNX1 interactome was predicted based on known interactions between orthologous proteins in yeast (*Saccharomyces cerevisiae*), nematodes (*Caenorhabditis elegans*), Drosophila (*Drosophila melanogaster*), and human (*Homo sapiens*; Geisler-Lee et al., 2007). To construct the interaction map, “At5g06140” was used as a string in the Arabidopsis Interactions Viewer^1^. The full list of genes encoding predicted SNX1-interacting proteins is available in **Supplementary Table S1**.

### Generation of Gene Coexpression Networks

**FIGURE 1:**
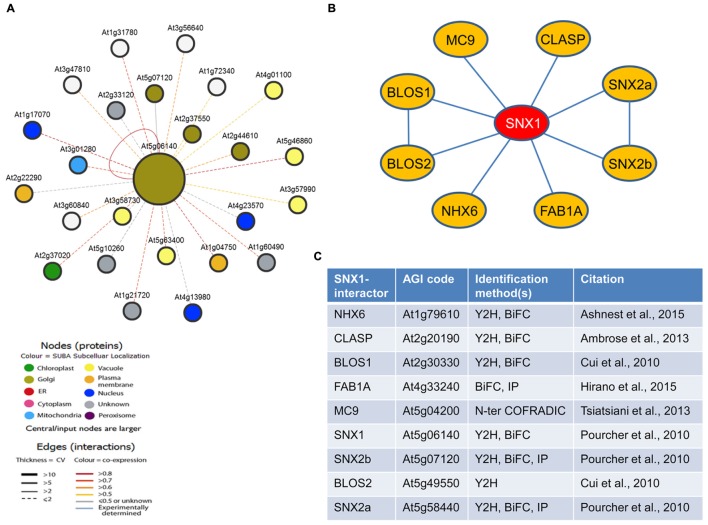
**Protein interactions of SNX1.** **(A)** Prediction-based SNX1-interaction map generated using Arabidopsis Interactions Viewer. **(B)** SNX1-interaction map based on literature data. Known interactions between BLOS1 and BLOS2, and SNX2a and SNX2b are also presented. **(C)** A list of the known SNX1-interacting proteins. The list includes the methods by which interaction was inferred, as well as the corresponding citation. Y2H, yeast two-hybrid screen; BiFC, bimolecular fluorescence complementation; IP, coimmunoprecipitation; N-ter COFRADIC, N-terminal combined fractional diagonal chromatography.

**FIGURE 2:**
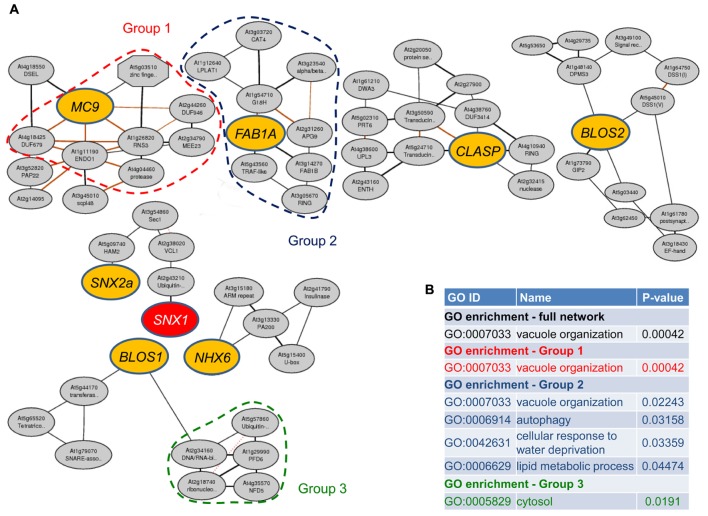
**SNX1 interactome-related coexpression networks.** **(A)** Genes encoding SNX1-interacting proteins (colored ovals) were used as a basis for the generation of the coexpression networks using the ATTED-II server. **(B)** Enriched gene ontology (GO) categories. The color code corresponds to the three groups of genes showing GO enrichment encircled with punctate lines in **(A)**.

The Arabidopsis Genome Initiative (AGI) numbers of the genes encoding the full SNX1 interactome, presented in **Figures 1B,C**, were used as a basis to build co-expression networks using the “Network Drawer” module of the online tool ATTED-II version 8.0^2^ (Aoki et al., 2016). The full list of the cluster-associated gene AGI numbers is available in **Supplementary Table S2**. Gene ontology (GO) category-enrichment data are available in **Figure 2B** and in **Supplementary Table S3**.

### Plant Material and Growth Conditions

Arabidopsis plants expressing *SNX1pro:SNX1-GFP* in *snx1-1* background (referred to as SNX1-GFP further in the text), a kind gift from Dr. Thierry Gaude (Jaillais et al., 2006), were surface sterilized in 70% ethanol for 3 min. The seeds were then plated on plates with Hoagland medium [1.5 mM Ca(NO_3_)_2_, 1.25 mM KNO_3_, 0.75 mM MgSO_4_, 0.5 mM KH_2_PO_4_, 50 μM KCl, 50 μM FeEDTA, 50 μM H_3_BO_3_, 10 μM MnSO_4_, 2 μM ZnSO_4_, 1.5 μM CuSO_4_, 0.075 μM (NH_4_)_6_Mo_7_O_24_, pH 5.8] supplemented with 1.4% plant agar (Duchefa). The plates with the seeds were stratified at 4°C for 2 days before transferring to a growth chamber and growing upright for 8 days. The incubation conditions were 16 h light at 21°C, 8 h darkness at 19°C. The seedlings were used for imaging.

### Confocal Microscopy

The seedlings were incubated in 10 mg/ml propidium iodide for 1 min, washed in distilled water and mounted on microscopic slides in liquid Hoagland medium. An LSM 780 (Zeiss) confocal microscope was employed for the study. For green fluorescent protein (GFP) visualization, excitation at 488 nm and detection between 505 and 545 nm were used. For propidium iodide, excitation at 561 nm and detection from 575 to 615 nm were used. Pinholes for both channels were set to 1 Airy Unit with optical slices equivalent to 0.8 μm. Images were recorded in a 1024 pixel format.

### Visualization of Root-Zone Gene Expression

Data from microarray analysis of root samples (Birnbaum et al., 2003; Nawy et al., 2005) was visualized using the ePlant browser^3^ (Fucile et al., 2011). The AGI numbers of the genes encoding the full SNX1 interactome, presented in **Figure 1C** were used as input.

### Hierarchical Clustering of Gene Expression Under Stress Conditions

The Genevestigator database^4^ (Hruz et al., 2008) was queried using the AGI numbers of the genes presented in **Figure 1C**. The selected stress conditions were the following: “response to auxin including natural variation”—AT-00104, AT-00164, AT-00165, AT-00167, AT-00226, AT-00257, AT-00407, AT-00543, AT-00655, AT-00658, and AT-00660; “heat stress”—AT-00026, AT-00120, AT-00129, AT-00387, AT-00402, AT-00439, and AT-00645; “iron deficiency”—AT-00286, AT-00333, AT-00348, AT-00449, and AT-00517. “Hierarchical clustering” tool was used to cluster both genes and conditions for similar patterns. Resulting trees represent Euclidean distance.

### Searches for Differential Protein and Phosphopeptide Abundance

Known peptides of SNX1 and the SNX1-interacting proteins were obtained from The Plant Proteome Database^5^ (Sun et al., 2009). The source publications were manually examined for cases of differential protein abundance when comparisons were made by the authors.

Experimentally identified phosphopeptides were recovered from the PhosPhAt 4.0 database^6^ (Durek et al., 2010). The full list of detected phosphopeptides is available in **Supplementary Table S5**. The source publications were manually examined for cases of differential phosphorylation when such comparisons were made by the authors.

## Results

### The Protein Interaction Map of SNX1 in Arabidopsis

We aimed to understand the regulatory events involved in modulating SNX1 activity under stress conditions. Therefore, our first goal was to identify the SNX1 protein environment. We applied two different approaches. First, a list of predicted SNX1 protein interactors was made, based on the homology of Arabidopsis proteins to known SNX1 interactors in other organisms (Geisler-Lee et al., 2007). The resulting 25 interactions included many intracellular trafficking-related proteins, such as syntaxins, subunits of the Exocyst complex, small GTPases, and microtubule-associated proteins (**Figure 1A**; **Supplementary Table S1**). Also included were a SNX1 self-dimerization, a hetero-dimerization with SNX2b and an interaction with the core retromer subunit VPS29. The majority of the interaction predictions, a total of 17 (68%), were based on yeast (*S. cerevisiae*) data. In addition, none of the interactions was seen in another of the organisms included in the study (**Supplementary Table S1**). This suggests that the SNX1 interactome might be largely species-specific.

In a second approach, we then screened the available literature for experimentally demonstrated SNX1 interactions in Arabidopsis. We found a total of nine documented interactions (**Figures 1B,C**), which included SNX1 and SNX2b but also another SNX, SNX2a. SNX1 was shown to be critical for the loading of SNX2a and SNX2b at the endosomal membrane (Pourcher et al., 2010). Two of the interactors, BLOS1 and BLOS2, are homologs of mammalian BLOC1-complex subunits (Cui et al., 2010). In mammals, the BLOC1–3 complexes are involved in protein sorting toward the lytic compartment (Rodriguez-Fernandez and Dell’Angelica, 2009). On the other side, the microtubule plus-end-localized protein CLASP was shown to bind and stabilize SNX1, and mediate SNX1-dependent recycling of the auxin transporter PIN1 (Ambrose et al., 2013). Two recent papers show the interaction with a sodium-proton antiporter NHX6 (Ashnest et al., 2015) and the 1-phosphatidylinositol-3-phosphate 5-kinase FAB1A. FAB1A was found to be essential for the recruitment of SNX1 to the endosomal membrane and the trafficking of PIN1 (Hirano et al., 2015). Interestingly, using a proteomics-based approach SNX1 was shown to be a target of the metacaspase-family protease MC9 (Tsiatsiani et al., 2013). MC9 gene is expressed very specifically in tracheary element cells destined to undergo programmed cell death and the MC9 protein is known to promote autophagy (Bollhoner et al., 2013; Escamez et al., 2016). It has to be added that *Brassica oleracea* SNX1 was shown to interact with the kinase domains of *Brassica* receptor kinases SRK_29_ and SFR1, and a kinase-dead form of the Arabidopsis CLV1 (Vanoosthuyse et al., 2003). At the same time, however, no interaction has been demonstrated between the Arabidopsis SNX1 and either the aforementioned receptor kinases or the two transporters, PIN2 and IRT1, whose recycling it is known to mediate.

In summary, only two of the nine experimentally demonstrated SNX1-interactors, SNX1 itself and SNX2b, have been predicted, underlining the specifics of plant SNX1 function. Therefore, in our further search for regulatory mechanisms involved in plant SNX-based protein sorting we concentrated our analysis on the list of experimentally demonstrated interactions (**Figure 1C**).

### Coexpression of Genes Encoding SNX1 Interactors

We performed an analysis of gene coexpression looking for potential hints to the processes involved in SNX1 regulation. With the exception of *SNX2b*, expression data for the other eight genes in the list in **Figure 1C** is available in public databases. The results, presented in **Figure 2A** and **Supplementary Table S2**, showed that a significant degree of coregulation of gene expression could be found only for *SNX1* and *SNX2a*. Each of the other six genes could be placed in a separate coexpression cluster, including *BLOS1* and *BLOS2* which are potential members of the same complex. This suggests that the amount of proteins available for interaction with SNX1 depends on a large variety of factors, potentially both the developmental program and the influence of external signals. Therefore, regulation on gene expression level may be a critical factor controlling the availability of SNX1 interactors in the cell, and thus affecting the composition, and the activity of the SNX1-containing complexes.

Among the genes in the coexpression clusters, at least six, At2g43210, At3g05670, At3g13330, At4g10940, At4g38600, At5g15400, and At5g57860 are predicted to have functions related to ubiquitination and protein degradation. This fact is especially intriguing considering that dynamic ubiquitination and deubiquitination events may be crucial for the sorting of endocytosed membrane proteins in the endomembrane system (Luschnig and Vert, 2014; Schuh and Audhya, 2014). We further explored the list of coexpressed genes for enrichment of GO categories. One category, “vacuole organization,” was significantly enriched (*P*-value 0.00042) in the whole data set (**Figure 2B**; **Supplementary Table S3**). Looking for GO enrichment within the single clusters, we could identify additional enriched categories with *P*-values lower than 0.05. These included “autophagy,” “lipid metabolic process,” and “cellular response to water deprivation” for the cluster that included the *FAB1A* gene. The “vacuole organization” category was enriched in two clusters, containing the *MC9* and *FAB1A* genes. An additional category, “cytosol,” could be identified as enriched with a *P*-value of 0.0191 in the *BLOS1*-containing cluster (**Figure 2B**; **Supplementary Table S3**). The genes representing the respective GO enrichment category are encircled by a punctate line with the respective color (**Figure 2A**).

Taken together, these data show a clear tendency for the involvement of different factors, including responses to external stress-related stimuli, for the regulation of the genes encoding the protein partners of SNX1. In addition, different genes might be targeted by different stimuli, resulting in specific changes in SNX1-mediated sorting.

### Root-Zone Expression of Genes Encoding SNX1 Interactors

**FIGURE 3:**
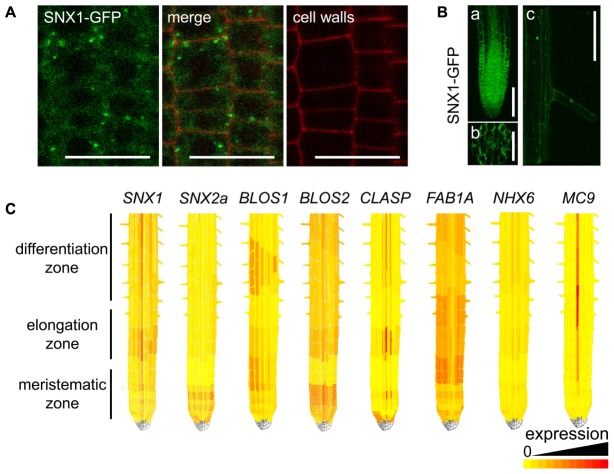
**Expression pattern of *SNX1* and genes encoding SNX1 interactors along the root.** **(A)** Subcellular localization of a complementing SNX1-GFP fusion in cells of the root meristematic zone. **(B)** Tissue-level expression of SNX1-GFP: **(a)** optical longitudinal section of the root meristematic and elongation zones; **(b)** optical cross-section of the region between the elongation and early differentiation zones; **(c)** a root hair cell. **(C)** Visualization of SNX1-interactor-encoding gene expression along the root using the ePlant tool. Relatively low expression is represented by a yellow color, high expression is shown in darker orange and red.

We investigated the presence of SNX1 interactors in zones of the root, which are known to require the function of SNX1 upon external stimuli. First, we used an Arabidopsis line expressing a SNX1-GFP fusion under the *SNX1* promoter to visualize the presence of SNX1 in different zones of the root. The fusion was shown to complement both the auxin- and iron deficiency-related *SNX1* loss-of-function phenotypes (Jaillais et al., 2006; Hanzawa et al., 2013; Ivanov et al., 2014). SNX1-GFP could be observed in the cytosol, as well as in punctate structures, corresponding to the sorting endosomes (Jaillais et al., 2008; **Figure 3A**). The majority of the GFP signal could be seen at the root tip, while in the basal direction the signal became weaker (**Figure 3Ba**). Despite that, SNX1-GFP was clearly detectable in the central cylinder and the epidermis of the early differentiation zone (**Figure 3Bb**), including the root hairs (**Figure 3Bc**). This localization pattern is consistent with the described functions of SNX1.

We then used the ePlant browser^7^ to visualize the expression of the genes encoding SNX1 interactors. Root expression was found for all genes, with the majority showing relatively high presence at the root tip and enrichments in specific zones (**Figure 3C**). Enhanced expression in the elongation zone could be seen for *SNX1*, *BLOS2*, *CLASP*, and *FAB1A*, while *BLOS1* was enriched in the meristematic-elongation zone transition and the differentiation zone. The complementary expression patterns of *BLOS1* and *BLOS2* in the meristematic and the elongation zone indicate that they either have diverse cell type-specific functions or they have a redundant function but are targeted by different regulatory inputs. *NHX6* showed relatively weak but uniform expression throughout the root. In comparison to all others, *MC9* showed a xylem-specific expression, in agreement with data from promoter–reporter fusion experiments (Bollhoner et al., 2013). Signal in other cells was low and there is a possibility that the protein is not synthesized there. Therefore, its influence on SNX1 might by strongly spatially specific.

The root gene-expression data shows that the SNX1-related genes have different expression patterns and is in agreement with the coexpression cluster analysis. It suggests that in the different zones of the root different protein partners will be available to form a complex with SNX1, and thus the SNX1 activity, may vary.

### Stress-Induced Changes of SNX1 Interactome-Related Gene Expression

**FIGURE 4:**
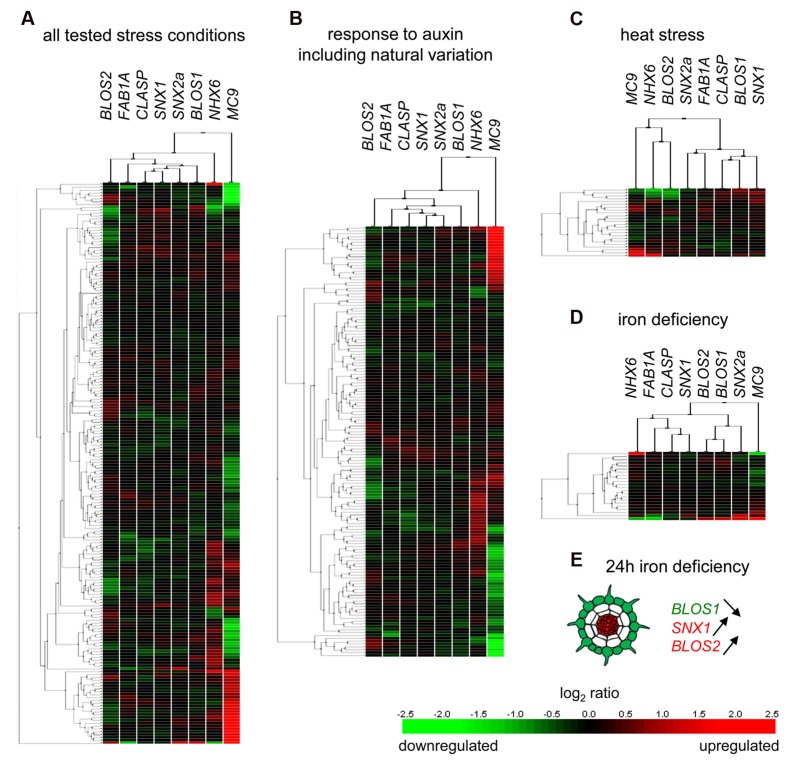
**Hierarchical clustering of SNX1-interactor gene expression in response to environmental inputs.** External inputs were chosen where plant responses require the function of SNX1. These include: **(A)** a combination of all stresses, **(B)** auxin application, including the responses of different Arabidopsis accessions, **(C)** heat stress, and **(D)** iron deficiency. **(E)** Radial-zone gene expression changes after 24 h of iron deficiency. Green color indicates downregulation in the performed comparisons, red color indicates upregulation. Panels **(A–D)** were generated using the Genevestigator tool.

Based on the finding that the availability of the SNX1-interacting partners is root-zone specific, we asked if this might also be influenced by environmental stress conditions. Since SNX1 is required for the regulation of transporter protein recycling upon cues, such as gravitropic stimulation, heat stress, and iron deficiency, we screened the Genevestigator database for comparative studies of the influence of auxin treatment, heat stress, or iron deficiency on gene expression in Arabidopsis roots or seedlings. We performed hierarchical clustering of gene expression among all selected treatments, as well as for each treatment type separately (**Figures 4A–D**). We could see that there were only limited gene expression changes, with the exception of the *MC9* and *NHX6* genes whose expression was most strongly affected. Under heat stress, *MC9* and *NHX6* clustered together with *BLOS2* (**Figure 4C**), while under all other stimuli *MC9* did not cluster with the rest (**Figures 4A,B,D**). When compared to each other, *NHX6* and MC9 showed very different expression patterns in response to the stimuli, which might partially reflect the cellular specificity of *MC9* expression. This result, however, also suggests that targeting these two genes is a possible mechanism of regulating the activity of SNX1. The similarities observed between the clusters of auxin treatment and iron deficiency stress are to be expected, as auxin is known to influence the expression of the iron uptake system and specifically the expression of the *IRT1* gene (Chen et al., 2010; Blum et al., 2014).

We screened microarray data for genes, related to the SNX1 interactome, that are regulated in a zone-specific manner under stress. Our criteria were a minimum of 1.5-fold induction or a minimum of 0.5-fold reduction compared to the control condition in the same zone. In the data presented by Dinneny et al. (2008), we found that a 24-h iron deficiency results in the downregulation of *BLOS1* in epidermis cells (**Figure 4E**). Since BLOS1 was shown to promote vacuolar targeting of cargo proteins (Cui et al., 2010), this result suggests a preferential recycling of endosome-located plasma membrane proteins under these conditions. Consistent with this, a SNX1-dependent IRT1 endosomal recycling in the epidermis has been shown to be crucial for IRT1 localization at the plasma membrane under iron deficiency (Ivanov et al., 2014). It has to be noted, however, that a role of BLOS1 in IRT1 trafficking has not yet been investigated. At the same time, the expression of *SNX1* and *BLOS2* is significantly upregulated in the central cylinder (**Figure 4E**) and also suggests enhanced stress-related trafficking. The upregulation of *SNX1* gene is consistent with the previously observed increased abundance of SNX1-GFP protein under iron deficiency (Ivanov et al., 2014).

Taken together, the data show that under stimuli external for the plant, there is a distinct transcription-level response that, in the case of iron deficiency, ensures cell-specific changes related to the composition of SNX1-containing protein complexes.

### Phosphorylation of SNX1-Interacting Proteins in Response to External Challenges

We investigated whether the changes of gene expression are also mirrored at protein level. We identified published peptides, corresponding to five of the proteins (**Supplementary Table S4**). Among the several comparisons in stress conditions, including abscisic acid (ABA) application and nitrogen starvation (see **Supplementary Table S4**) there were no differences reported for any of the SNX1-interacting proteins, whenever these proteins were identified. However, it is still possible that such differences in protein abundance will be detected in future comparisons. For SNX1, for example, we have recently reported that its protein levels are increased under iron deficiency conditions.

We then looked for potential post-translational modifications that target members of the SNX1 interactome. SNX1 itself is a known phosphorylation target. It has been demonstrated that application of auxin enhances the phosphorylation of SNX1 at serine 16 and that this modification is important for lateral root formation (Zhang et al., 2013). We screened the PhosPhAt 4.0 database^8^ (Durek et al., 2010) for all instances of identified phosphopeptides in SNX1 interactors. We found phosphopeptides corresponding to seven of the proteins in the list (**Table 1**; **Supplementary Table S5**). Most of these were identified in multiple studies, only for MC9 a single peptide was reported. We then screened the literature for changes in the phosphorylation status under external challenge. We found that differential phosphorylation has been observed for three of the proteins, CLASP, FAB1A, and SNX1 in a total of four different studies (**Table 1**). CLASP phosphorylation was downregulated by osmotic stress (Xue et al., 2013), while FAB1A phosphorylation was upregulated in the *rcn1-1* mutant, lacking the regulatory subunit A1 of protein phosphatase 2A (PP2A), compared to wild type (Yang et al., 2013). The *rcn1-1* mutant is known to exhibit auxin transport and other phytohormone-related phenotypes (Saito et al., 2008). As mentioned above, SNX1 phosphorylation increases in response to auxin (Zhang et al., 2013). However, the same serine on position 16 was shown to be dephosphorylated under ionizing radiation (Roitinger et al., 2015).

**Table 1:** Differential phosphorylation of SNX1-interacting proteins.

SNX1-interactor	AGI code	Phosphorylation found	Differential phosphorylation
NHX6	At1g79610	Yes	–
CLASP	At2g20190	Yes	Downregulated by osmotic stress (Xue et al., 2013)
BLOS1	At2g30330	No	–
FAB1A	At4g33240	Yes	Upregulated in *rcn1-1* compared to wild type (Yang et al., 2013)
MC9	At5g04200	Yes	–
SNX1	At5g06140	Yes	Upregulated by auxin (Zhang et al., 2013); downregulated under ionizing radiation (Roitinger et al., 2015)
SNX2b	At5g07120	Yes	–
BLOS2	At5g49550	No	–
SNX2a	At5g58440	Yes	–


*Known cases of phosphorylation of the SNX1-interacting proteins are listed together with the respective cases of differential phosphorylation and the corresponding references. A list of references (PubMed ID) showing the phosphorylation identification and the corresponding detected peptides is available in **Supplementary Table S5**.*

These data show that certain SNX1-interacting proteins are selectively targeted for phosphorylation and, therefore, different external stimuli might target SNX1 activity in a direct or indirect manner. Interestingly, the lists of targets for transcriptional regulation and phosphorylation are complementary. This demonstrates how a great variety of inputs can be accommodated through differential availability of subunits and their alternation between active and inactive state in order to coordinate protein recycling in a cell-specific manner.

## Discussion

Endocytosed plasma membrane proteins might be targeted for degradation or be recycled at the sorting endosome and reused. Presence of many Arabidopsis plasma membrane proteins has been detected in the sorting endosome (Jaillais et al., 2006, 2008; Ivanov and Gaude, 2009; Ivanov et al., 2014). The SNX1 protein has been implicated in the retrieval of plasma membrane transporters, such as PIN2 and IRT1 from the degradation pathway. We generated a complete list of known SNX1-interacting proteins and used publicly available data to analyze their transcriptional and post-translational regulation in response to environmental stresses.

### Transcription as a Means to Regulate Vesicle Trafficking

We showed that the genes encoding SNX1-interacting proteins differ substantially in their expression levels and cellular expression patterns, suggesting that the composition of SNX1-containing protein complexes is different between one cell type and another. Intriguingly, we saw that external signals in the form of heat, application of auxin or iron limitation, known to trigger SNX1-dependent responses, result in the differential regulation of a few genes, rather than the whole interactome. Analysis of expression data under iron deficiency stress shows that these changes are root-zone specific and are consistent with rebalancing SNX1 activity in different root-cell types. Transcriptional regulation emerges as a mechanism for regulating endomembrane trafficking in all organisms. In mammals, it was demonstrated as a key prerequisite for the function of Rab-family small GTPases upon pathogen infection (Lizundia et al., 2007; Pei et al., 2012). Mammalian genes encoding lysosome biogenesis proteins share a common functional *cis*-regulatory motif, a target of the transcription factor TFEB (Sardiello et al., 2009). In plants, there are also examples of transcriptional regulation of endomembrane trafficking. The gene encoding the v-SNARE protein VAMP711 is downregulated under salt stress, which leads to enhanced salt tolerance, as seen by using antisense lines and T-DNA insertion mutants (Leshem et al., 2006). Coexpression analysis of iron-deficiency marker genes expressed in leaves also identified a cluster of genes which included many trafficking components, such as a RAB GTPase, a Rho guanidine exchange factor and a Rho GTPase activating protein, among others (Ivanov et al., 2012). Our coexpression analysis shows a prominent enrichment of the “vacuole organization” GO category. Since the vacuole is the plant cell’s lytic compartment, this finding suggests that a similar mechanism of transcriptional regulation might also exist in plants. Therefore, activation or inhibition of gene expression might be an important component in the regulation of vesicle trafficking when rebalancing plant homeostasis under stress.

### Regulation of SNX1 and Its Interacting Partners Under Stress

Our data show that environmental stress has a complex impact on the regulation of the availability and the state of the proteins interacting with SNX1. We have discovered a general tendency of transcriptional regulation and protein phosphorylation affecting the analyzed proteins in a complementary pattern in response to stress. Thus, the majority of proteins significantly affected on the level of mRNA abundance, such as NHX6 and MC9, show no differential phosphorylation or even no phosphorylation at all. At the same time, the expression levels of the genes encoding *CLASP* and *FAB1A* show no major alterations but the proteins undergo differential phosphorylation under different stresses. SNX1 itself seems to be an exception, as it shows both significant transcriptional and phosphorylation changes. A closer look shows that these are stress-specific alterations and they are also complementary. The levels of SNX1 in response to iron deficiency are upregulated in the cells of the central cylinder. These data are consistent with the previously reported observation of enhanced protein abundance under these conditions (Ivanov et al., 2014) but no significant differences in SNX1 phosphorylation were observed under this stress (Lan et al., 2012). Upon auxin treatment, the phosphorylation of serine 16 is enhanced which stimulates the activity of SNX1 (Zhang et al., 2013), despite the fact that no major gene expression alterations can be detected. Altogether the data shows that the presence and activity of SNX1 are stress-regulated.

An intriguing finding was the contrasting regulation of BLOS1 and BLOS2. Despite the fact that the two are members of the same complex, BLOC1, the genes encoding them display a dramatically different expression pattern. In the root zones, the expression of the two is almost opposite to each other. In zones where BLOS1 shows enhanced mRNA abundance, BLOS2 expression is low, and the opposite is true for zones with low BLOS1 expression. This situation is mirrored in response to stress conditions, where the two genes tend to cluster away from each other. Under iron deficiency, the two also show opposite responses, however, the regulatory effect can be observed in different radial zones of the root, namely upregulation of BLOS2 in the central cylinder and downregulation of BLOS1 in the epidermis. It has to be mentioned that no phosphorylation has been detected for either BLOS1 or BLOS2. While other potential modifications cannot be excluded, this implies that the activity of the BLOC1 complex might be balanced through targeted cell-specific depletion or enhancement of subunit abundance. This has also implications for the regulation of SNX1. The phenotypes of the *BLOS1/BLOS2*-suppressed plants are opposite to those of *snx1* mutants, including the regulation of PIN proteins (Cui et al., 2010). Thus, it may be speculated that the SNX1–BLOS interaction inhibits SNX1, sequestering it from the active recycling complex and keeping it as a ready, temporarily inactive pool at the endosomal membrane. In any case, the interaction might serve for balancing the processes of recycling and vacuolar targeting and the enhanced or diminished presence of BLOS1 and 2 potentially affects SNX1-mediated protein recycling. The observed regulation of the genes encoding these proteins in radial root zones is, therefore, consistent with the known effects of SNX1 on IRT1 trafficking in the root epidermis, and with an enhanced trafficking in the central cylinder, respectively.

Due to the current lack of data, it is difficult to predict the role of protein phosphorylation for the activity of CLASP and FAB1A. Nevertheless, their significant response to osmotic stress and *rcn1-1*-associated defects, respectively, indicates that these proteins are targets of the cellular signaling systems. In the context of the regulation of SNX1-mediated trafficking, it is tempting to speculate that differential phosphorylation of CLASP and FAB1A may regulate the association of SNX1 at the endosomal membrane and, therefore, protein recycling. Thus, it will be of great interest to understand the role of CLASP and FAB1A phosphorylation and analyze the performance of SNX1 in plants expressing constitutively dephosphorylated or phosphomimicking versions of these two proteins.

### Interactions and Inputs Regulating SNX1 Activity

**FIGURE 5:**
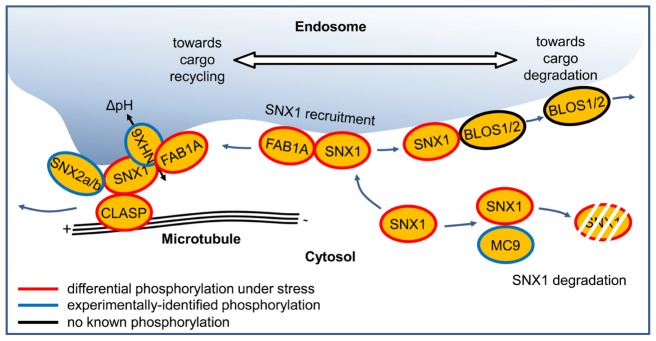
**A model of the events involved in SNX1-mediated protein recycling.** The surface of the endosome in an idealized cell containing all SNX1 interactors is represented. The “plus” and the “minus” ends of a microtubule are indicated. Proteins are represented as oval shapes bordered by black lines (when no phosphorylation has been experimentally demonstrated), blue lines (when phosphorylation was observed), and red lines (when differential phosphorylation was shown under stress).

The participation of the SNX1-interacting proteins in the SNX1-mediated sorting process is illustrated in **Figure 5** for an idealized situation, where all currently known components are readily available. SNX1 protein has dual localization, in the cytoplasm and at the endosomal membrane (Jaillais et al., 2006). Under certain conditions, cytoplasmic SNX1 can become a target of MC9 in tracheary elements (Tsiatsiani et al., 2013), or of other proteases which may mediate its degradation in different cell types. SNX proteins are recruited to the endosomal membrane via their PHOX (NADPH Phagocyte Oxidase) homology domain (Teasdale and Collins, 2012). There is evidence that SNX1 binds phosphatidylinositol-3-phosphates (Pourcher et al., 2010; Simon et al., 2014). The 1-phosphatidylinositol-3-phosphate 5-kinase FAB1A, through a direct interaction, was shown to be the prerequisite for SNX1 recruitment to the endosome (Hirano et al., 2015). Therefore, SNX1 might also bind phosphatidylinositol-3,5-bisphosphate, which is a product of FAB1A enzymatic activity. Recently it was shown that the lipid composition of the endosomal membrane affects significantly SNX1-mediated trafficking (Chu et al., 2016). Once at the endosomal membrane, SNX1 can participate in complexes with either the vacuolar targeting-promoting BLOC complex subunits BLOS1 and 2 or with the recycling-promoting components FAB1A, SNX2a/b, CLASP, and NHX6 (Cui et al., 2010; Pourcher et al., 2010; Ambrose et al., 2013; Ashnest et al., 2015). As discussed above, the SNX1–BLOS may be involved in balancing between recycling and vacuolar targeting. On the other side, the role of SNX1 in recycling is well documented. The SNX1-mediated recruitment of a SNX2 protein to the membrane is required, since as a heterodimer their predicted function is to induce membrane curvature and subsequent tubulation (Pourcher et al., 2010; Zelazny et al., 2012). The activity of NHX6 depends on the function of its C-terminus which also contains the SNX1-interaction site (Ashnest et al., 2015). Finally, through its interaction with SNX1, CLASP mediates the contact between the microtubule plus-end and the forming transport vesicle (Ambrose et al., 2013; **Figure 5**). Thus, changing the availability of the different components can trigger a change in the functionality of SNX1.

### Perspectives

Varying environmental conditions result in selective changes of the transcriptional behavior of genes encoding SNX1 interactors or the post-translational regulation through phosphorylation of their protein products. In the context of SNX1-mediated protein trafficking, these complementary events might represent an efficient and versatile way of regulating the activity of SNX1 in response to external stimuli. The findings presented in this work provide a basis for studying the mechanistic details of the regulatory events involved in protein trafficking under stress. In the case of SNX1, several questions will need to be tackled in order to be able to outline a plausible regulatory model. These include identifying different SNX1-containing complexes and their exact composition. These complexes should be analyzed in a cell-specific manner, taking into account their dynamics in response to stress. The aim should be to expand the SNX1-interactors network beyond the direct SNX1 protein interactions, an approach which will potentially reveal novel aspects into how stress responses are integrated in the developmentally regulated trafficking. An intriguing recent finding in this direction was the observation that SNX1 coimmunoprecipitates with the small GTPase ARA7 (Heard et al., 2015).

## Author Contributions

RI designed the concept. RI and TB performed analysis and discussed results. RI wrote the manuscript.

## Conflict of Interest Statement

The authors declare that the research was conducted in the absence of any commercial or financial relationships that could be construed as a potential conflict of interest.

## Footnotes

1 http://bar.utoronto.ca/interactions/cgi-bin/arabidopsis_interactions_viewer.cgi

2 http://atted.jp/top_draw.shtml#NetworkDrawer

3 https://bar.utoronto.ca/eplant/

4 https://genevestigator.com/gv/

5 http://ppdb.tc.cornell.edu/

6 http://phosphat.uni-hohenheim.de

7 https://bar.utoronto.ca/eplant/

8 http://phosphat.uni-hohenheim.de
